# Validating an assessment and feedback instrument for use in dietetics education: construct validity of the mini clinical evaluation exercise (Mini-CEX)

**DOI:** 10.1186/s12909-025-08255-8

**Published:** 2025-12-12

**Authors:** Anna Kleppe Moe, Helene Dahl, Monika Kvernenes, Aslaug Drotningsvik, Hanne Rosendahl-Riise

**Affiliations:** 1 Centre for Nutrition, Department of Clinical Medicine, University of Bergen, Bergen, Norway; 2 Centre for Medical Education, Faculty of Medicine, University of Bergen, Bergen, Norway; 3 Department of Clinical Medicine, University of Bergen, Bergen, Norway

**Keywords:** Mini-CEX, Validity, Construct validity, Formative assessment, Feedback, Dietetics education, Health professions education, Clinical placement, Workplace-based assessment

## Abstract

**Background:**

Dietetics education lacks standardised tools for formative assessment and feedback in clinical placement for students, which impairs learning outcomes. The Mini Clinical Evaluation Exercise (Mini-CEX) is a seven-item instrument designed for clinical supervisors’ direct observation and assessment of student performance on six clinical competencies plus overall competence. It has been validated in contexts of several disciplines, but not in dietetics education. In this study we aim to assess the construct validity of Mini-CEX for this context.

**Methods:**

We developed three videotapes portraying dietetics students carrying out patient consultations in secondary health care. An assessment panel of dietitians reached consensus regarding the level of competence portrayed by each student. Clinical dietitians were then invited to watch the videos individually in randomised order and assess the students’ clinical competencies using the Mini-CEX instrument. Construct validity was investigated by analysing agreement between assessment panel and participants’ scores, interrater reliability, and rank correlation between competencies.

**Results:**

Clinical dietitians (*n* = 65) working in secondary health care in Norway participated in the study. On a scale of 1–9, the assessment panel’s scores on the three students’ overall clinical competence were 2, 5 and 7, participants mean scores were 1.9, 4.9 and 7.6, respectively. The proportion of participants’ scores agreeing with the assessment panel scores ranged from 63 to 97% on the six competencies and overall competence. Interrater reliability between participants’ scores was substantial (Krippendorff’s alpha = 0.772). There were significant positive rank correlations between the competency variables in all videos (Kendalls’ tau = 0.49–0.86, *p* < 0.0001).

**Conclusions:**

Our results indicate that participants were able to discriminate between dietetics students’ levels of clinical competence using the Mini-CEX instrument in agreement with an assessment panel. The observed agreement and interrater reliability of the Mini-CEX scores are in favour of its construct validity in dietetics education. A valid observation and assessment using Mini-CEX constitutes an important step of the formative assessment process. Consensus amongst assessors and training in how to provide feedback is needed to ensure successful implementation.

**Supplementary Information:**

The online version contains supplementary material available at 10.1186/s12909-025-08255-8.

## Introduction

Though pivotal to health professions students’ education and professional development, feedback based on direct observation of their clinical skills can be scarce due to workload demands and priorities in the clinic [1, 2]. This rings true for a variety of health personnel, clinical dietitians included [3], and is likely a precursor for students’ dissatisfaction with current feedback practices [4]. As a relatively new discipline of authorised health personnel with fewer graduates compared to medicine, dentistry, and nursing, the field of dietetics also suffers from a lack of well-established, discipline-specific, validated tools to enhance these processes [3, 5]. The majority of clinical dietitians will be asked to take on the role as a supervisor for dietetics students, without educational training, because supervision resources are often scarce [3, 5]. Though unintended, lack of tools and training for clinical supervisors can accumulate in missed learning opportunities for dietetics students and adversely impact the quality of clinical placement, deviating from desired placement standards [6, 7].

Dietetics-specific assessment and feedback instruments have been developed, but to our knowledge they have been little feedback-oriented, broadened the scope beyond direct observation by including aspects of preparation and documentation, or limited the assessment to only specific aspects of clinical competence [8–10]. Despite existing tools, in a 2024 scoping review, Knight et al. [11] still encouraged future development of validated dietetics-specific communication skills assessment tools. At the same time, there have been calls to move away from an overly objective and quantitative view of student assessment based in a psychometric tradition, as these approaches rely on isolated data points from single tools [12, 13]. With the emergence of programmatic assessment in health professions education (HPE) there has been a growing emphasis on a more holistic approach to assessment and feedback, including frequent assessments *for* learning that integrate both quantitative and qualitative information [14]. In response, initiatives have been launched to implement similar approaches specifically within dietetics education [15]. Mini Clinical Evaluation Exercise (Mini-CEX) is a promising tool to be utilised as one of multiple valuable data points, in such a holistic approach to workplace-based assessment of clinical competence in dietetics education.

The Mini-CEX is a seven-item instrument, widely recognised as a formative assessment and feedback tool in *medical* education [16]. It was developed to be a succinct, reliable and feedback-oriented instrument in the supervision of HPE students to assess skills needed in patient encounters, such as clinical communication skills, professionalism, and clinical reasoning [16, 17]. The assessment using Mini-CEX is conducted by a supervisor directly observing the student during a clinical encounter. The supervisor scores the student’s performance on a scale from 1 to 9 (assessment categories: 1–3 = unsatisfactory, 4–6 = satisfactory, 7–9 = superior) on six clinical competencies which all have a corresponding descriptive anchor on what they entail. Additionally, one overall clinical competence score is given, and an open field allows for comments on prominent strengths and specific suggestions for improvement. The observation is expected to take about 15 min and should ideally be repeated for several clinical encounters and settings with different assessors to provide a broad overall assessment of the student’s clinical competence [18]. Immediately after the observation, the supervisor and student have a structured feedback conversation based on the scores and comments, which – if adequately incorporated – has the potential to enhance the student’s future performance and professional development through assessment *for* learning [19, 20]. The Mini-CEX structure helps the supervisor to focus their attention on specific areas while observing and to express their feedback in a clear and concise manner naming prominent strengths, weaknesses, and creating an action plan together with the student [18]. Globally, Mini-CEX has been adapted and validated across a variety of medical settings, specialties and levels of training [18], and also in other disciplines, such as dental and nursing educations [21, 22]. To our knowledge, is has been adapted, but not yet validated, in the context of dietetics education [23].

Within the context of assessing *medical* students and residents the construct validity of Mini-CEX has been supported by demonstrating how students obtain higher scores by assessors when they are at higher levels of their studies or by increasing scores over time [24–26]. Criterion validity has been supported by Mini-CEX scores’ correlations with other measures of clinical competence as a predictive measure [26–29], and the feasibility and acceptability of the instrument has been supported across a range of medical specialties and contexts [25, 30–32].

In order to implement Mini-CEX for use in a new field of HPE, e.g. dietetics education, adaptation of the instrument must be followed by demonstration of acceptable validity for this particular context. Construct validity – whether the instrument measures the construct it is intended to – is a crucial component of overall validity [33]. For the Mini-CEX instrument, construct validity has been defined as the Mini-CEX scores’ “*ability to differentiate between levels of performance*” and ”*discrimination between competency levels*” [24, 34, 35]. The construct in question being clinical competence, composed of the six competencies plus overall competence, as they are defined in their corresponding descriptive anchors in the Mini-CEX instrument. Being a crucial component of any validity argument and a logical first step, for this study we aimed to evaluate the construct validity of the Mini-CEX instrument in assessing the clinical competence of *dietetics* students in clinical placement.

## Methods

### Context

The present study was conducted in Norway, where the dietetics education is currently held by three Higher Education Institutions (HEIs). It is either structured as a bachelor’s degree in nutrition followed by a master’s degree in dietetics or as an integrated master’s degree of five years, equivalent to 300 European Credit Transfer and Accumulation System (ECTS). In Norway, completion of this dietetics education leads to a protected title and authorisation as health personnel, in line with the European Federation of the Associations of Dietitians’ (EFAD) definition of a dietitian [36], with qualifications to work in both clinical and non-clinical settings. Following national regulations, students are required to complete a total of 14 weeks of clinical placement, six of these in secondary health care in their final year [37]. During this six-week placement, students are assigned one or more clinical dietitians as supervisors and are on several occasions observed directly performing patient consultations, in an increasingly independent manner. In this regard, Mini-CEX was adapted to the Norwegian dietetics field in 2022 [23] and has since been promoted as a suitable assessment and feedback instrument for clinical dietitians in supervising students. The instrument is increasingly being implemented in the formative assessment of dietetics students at the master level and is to be used in alignment with existing national competency standards for dietetics [37].

### Study design

For this quantitative validation study, we developed three videotapes with actors portraying fifth-year master students in dietetics carrying out patient consultations in secondary health care. First, an assessment panel watched the videos, assessed the students’ clinical competence using the Mini-CEX instrument, and established reference scores. We then invited clinical dietitians to watch the videos and assess the three students using Mini-CEX. The completed Mini-CEX forms were collected and scores were analysed. The study is undertaken from a post-positivist research orientation, meaning we seek evidence of truth through quantitative methods, but acknowledge the methods’ limitations and that they ultimately cannot fully capture the truth [38, 39].

### Data collection

#### *Videotapes*

In spring 2023, three scripted videotapes were recorded with actors portraying fifth-year master students in clinical placement depicting different levels of clinical competence in doing patient consultations, a method adapted from others [24, 32, 34]. The scripts were written by the main author (AKM: dietitian and HPE researcher), then reviewed and revised iteratively in collaboration with two of the co-authors (MK: HPE researcher, HRR: dietitian, nutrition researcher and educator). Script development was based on the descriptive anchors of “superior performance” for each competency in the Norwegian Mini-CEX for dietetics [23] as well as firsthand clinical experience by authors. The three scripts were written to demonstrate three levels of clinical competence: unsatisfactory, satisfactory, and superior, predominantly within the competencies *clinical communication skills*, *clinical reasoning*, and *professionalism*. The performance levels were based on the expected performance level of master students in dietetics, in the second half of their six-week final clinical placement.

The videotapes represented three common cases from the clinic, both inpatient and outpatient settings: The first video (hereafter referred to as “Overweight”) represented a middle-aged woman with previous breast cancer and current overweight (outpatient). The second video (hereafter referred to as “Malnourishment”) represented an elderly man with declining cognition and underweight at nutritional risk (inpatient). The third video (hereafter referred to as “Diabetes”) represented a middle-aged man with recently diagnosed type 2 diabetes mellitus and overweight (outpatient). We hired six actors to portray the three students and three patients (hereafter referred to as “students” and “patients”), and final video durations were 10, 12, and 14 min.

#### *Assessment panel*

Prior to the data collection, we assembled an assessment panel to score the students’ performance using Mini-CEX and reach consensus regarding the clinical competence depicted in the videos. The assessment panel consisted of four dietitians: two with formal training in clinical supervision and 10–20 years of clinical experience which has included supervising and assessing master students in clinical placement, both with and without Mini-CEX. The remaining two were among the authors (AKM, HRR), also dietitians with clinical and supervisory experience < 5 years, but currently working in academia. Participants of the panel watched the three videos individually in randomised order, whilst scoring the students in the videos on all competencies. Afterwards, individual scores were compared and discussed until the panel reached a consensus as to the level of performance depicted by the students. The panel confirmed that the cases, patients and students in the videos were realistically portrayed in accordance with the clinic. The assessment panel’s consensus was set as the reference scores in the validation process.

#### *Study participants*

Clinical dietitians working in secondary health care from all health regions in Norway were invited to participate in the study, mainly via email to department leaders who further distributed the information to their staff. Participation was encouraged by highlighting the educational value of participating, but no incentive was provided. Non-respondents were prompted twice with additional emails.

Data collection took place in 14 data collection sessions, with AKM physically present in five of these. Due to significant geographical distances, AKM instructed participants through digital Microsoft Teams meetings for the remaining nine sessions, with study participants sitting together physically or individually online. Participants were introduced to the research project as well as briefly how Mini-CEX is to be used by a supervisor in direct observation of their student. Then, participants anonymously, with assigned ID-numbers, completed an online seven-item background survey regarding work experience, supervisory experience and familiarity with Mini-CEX. Afterwards, they watched the videos individually in randomised case sequence while assessing each student’s performance using Mini-CEX. The participants were instructed to score the three students on all six competencies plus overall competence from 1 to 9, while leaving open the rubrics for comments on strengths and suggestions for improvement. The type of video was identified in the Mini-CEX by “Overweight”, “Malnourishment” and “Diabetes”, and Mini-CEX results were matched with background survey responses by the participants’ ID numbers. To provide an equal frame of reference for their specific context and supervisory practice, participants were informed that, according to university guidelines, scoring a student 1–3 on any of the categories at this intended time of their clinical placement, would be the equivalent to a report of concern to the placement coordinator at the university. Participants were told to not interact with each other until all had completed all three Mini-CEX forms. Completion – including instructions, viewing and scoring of the videos – took approximately 1–1.5 h.

### Data analyses

In assessing validity, single measures provide only limited information, not suitable for a detailed impression of an instrument [40]. Therefore, to assess aspects of the construct validity of the Mini-CEX in this setting, the data were analysed in various ways to enable a broader interpretation of the collected data. (1) Interobserver agreement, in the form of percent agreement [41], is reported to demonstrate the proportion of participants’ scores which matched the assessment panel’s scores, both exact score agreement and score agreement within the same assessment category (1–3 = unsatisfactory, 4–6 = satisfactory, 7–9 = superior). (2) Krippendorff’s alpha was applied to assess the interrater reliability corrected for chance agreement among the participants. Alpha coefficients were interpreted in accordance with guidelines of Landis and Koch [42] supported by Hughes [43]: α ≤ 0.2 indicating slight agreement, 0.2 < α ≤ 0.4 indicating fair agreement, 0.4 < α ≤ 0.6 indicating moderate agreement, 0.6 < α ≤ 0.8 indicating substantial agreement and α >0.8 indicating near-perfect agreement. (3) To determine the rank correlation between the seven competency scores, Kendall’s tau was performed for each video. Correlation coefficients 0.1–0.3 were considered low, 0.3–0.5 were considered medium, 0.5–0.7 were considered high, and 0.7–1.0 were considered very high [44]. Tests were chosen based on the categorical and ordinal nature of the variables. Statistical analyses were performed in RStudio version 4.3.1, findings were considered statistically significant when *p* ≤ 0.05. Descriptive statistics are presented as counts and percentages.

### Ethical considerations

The current study was approved by Sikt – Norwegian Agency for Shared Services in Education and Research, number 686615. All actors and all participants signed forms of voluntary informed consent prior to participation. After data collection was complete, the respondents were only identifiable through an identification key between individual ID numbers and names.

## Results

Clinical dietitians (*n* = 65) from ten hospitals and all four health regions in Norway participated in the study, with a majority from west and east of the country where most positions for clinical dietitians are held. The sample population consisted of predominantly young women, most with no formal training in supervision and no or little experience with Mini-CEX. All demographic variables are presented in Table 1.

**Table 1:** Descriptive data of sample population of clinical dietitians (*n* = 65)

Variable		*n* (%)
Gender		
	Female	64 (98)
	Male	1 (2)
Age		
	< 25 years	2 (3)
	26–35 years	31 (48)
	36–45 years	10 (15)
	46–55 years	15 (23)
	> 55 years	7 (11)
Health region		
	West	31 (48)
	Mid	1 (1)
	North	7 (11)
	South-East	26 (40)
Work experience as a dietitian		
	< 2 years	9 (14)
	2–4 years	13 (20)
	5–7 years	10 (15)
	8–10 years	5 (8)
	> 10 years	28 (43)
Formal training in supervision		
	Yes	15 (23)
	No	40 (77)
Number of master students supervised in clinical placement		
	0	14 (21)
	1–3	18 (28)
	4–6	13 (20)
	7–9	4 (6)
	≥ 10	16 (25)
Experience with Mini-CEX		
	None	34 (52)
	Some	30 (46)
	Extensive	1 (2)

*Mini-CEX Mini Clinical Evaluation Exercise*

On the scale of 1–9, the participants’ mean Mini-CEX scores for the overall clinical competence were 1.9 for the Overweight video, 4.9 for the Malnourishment video, and 7.6 for the Diabetes video (Table 2). These results matched the performance levels the video scripts were intended to demonstrate, equivalent to unsatisfactory, satisfactory and superior overall performance, respectively.

**Fig. 1:**
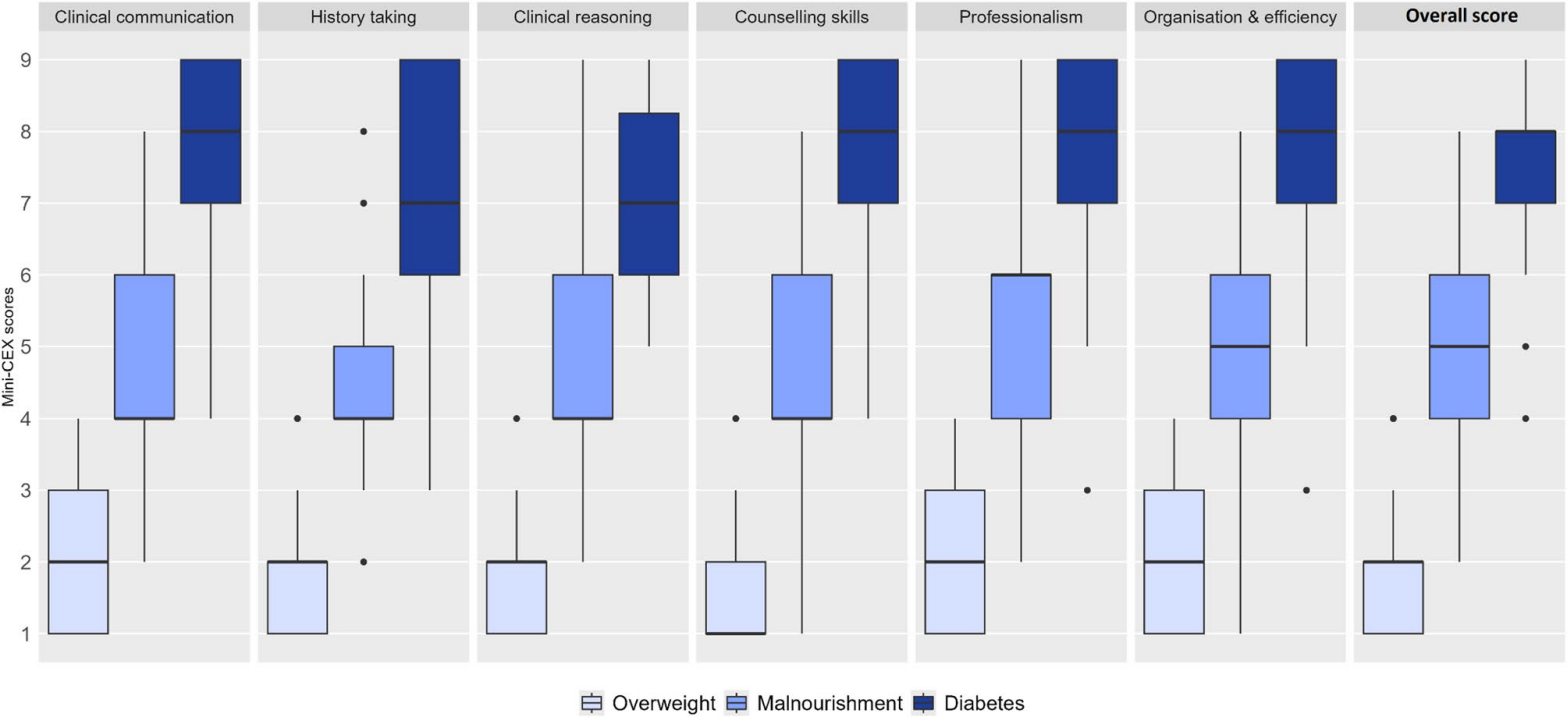
Boxplots of Mini-CEX scores for student performance in the three videos. Mini-CEX scores 1–3 indicate unsatisfactory performance, 4–6 satisfactory performance, 7–9 superior performance. Clinical communication; clinical communication skills, overall score; overall clinical competence

1) Exact percent agreement between participants’ Mini-CEX scores and the assessment panel consensus scores for all competencies on all videos ranged from 15 to 27%. When the participants’ scores of 1–9 were collapsed into the assessment categories (1–3 = unsatisfactory, 4–6 = satisfactory, 7–9 = superior), higher agreement ranging from 63 to 97%, was observed, with highest agreement on all competencies on the student’s performance in the Overweight video (Table 2).

2) The Krippendorff’s alpha, as a measure of interrater reliability and chance-corrected agreement, was 0.772 (95% CI: 0.629–0.796) for the entire dataset, indicating substantial agreement on the depicted level of competence in the three videos. There was a wide range in scores on all competencies in the Malnourishment video compared to the two others, ranging from “unsatisfactory” to “superior” (Fig. 1).

3) Kendall’s tau correlations for the Overweight video were between 0.49 and 0.83, for the Malnourishment video between 0.49 and 0.80, and for the Diabetes video between 0.57 and 0.86, indicating mostly high and very high positive correlations between the competency variables in all videos. All correlations were significant (*p* < 0.0001), and there were no observed differences between correlations for the three highlighted competencies (*clinical communication skills*,* clinical reasoning*,* professionalism*) and the remaining competencies.

**Table 2:** Participants’ (*n* = 65) mean Mini-CEX scores and percent agreement with assessment panel on student video performance

Video	Competence	Assessment panel score	Participant score, mean (95% CI)	Exact score agreement, *n* (%)	Assessment category* agreement, *n* (%)
Overweight	Clinical communication skills	2	2.0 (1.8–2.2)	21 (32.3)	62 (95.4)
	History taking	3	1.9 (1.7–2.1)	13 (20)	62 (95.4)
	Clinical reasoning	2	1.8 (1.6–2.0.6.0)	23 (35.4)	64 (98.5)
	Counselling skills	2	1.6 (1.4–1.8)	15 (23.1)	63 (96.9)
	Professionalism	2	2.2 (1.9–2.4)	24 (36.9)	54 (83.1)
	Organisation and efficiency	2	1.9 (1.7–2.2)	20 (30.8)	59 (90.8)
	*Overall clinical competence*	*2*	*1.9 (1.7–2.1)*	*27 (41.5)*	*61 (93.8)*
Malnourishment	Clinical communication skills	5	4.7 (4.4–5.1)	14 (21.5)	46 (70.8)
	History taking	5	4.6 (4.2–4.9)	11 (16.9)	46 (70.8)
	Clinical reasoning	4	4.8 (4.4–5.1)	22 (33.8)	46 (70.8)
	Counselling skills	5	4.8 (4.4–5.1)	14 (21.5)	44 (67.7)
	Professionalism	5	5.4 (4.9–5.8)	9 (13.8)	41 (63.1)
	Organisation and efficiency	5	5.2 (4.8–5.5)	18 (27.7)	44 (67.7)
	*Overall clinical competence*	*5*	*4.9 (4.5–5.2)*	*16 (24.6)*	*45 (69.2)*
Diabetes	Clinical communication skills	7	7.5 (7.2–7.9)	10 (15.4)	49 (75.4)
	History taking	7	7.4 (7.0–7.7.0.7)	16 (24.6)	47 (72.3)
	Clinical reasoning	7	7.4 (7.1–7.7)	16 (24.6)	47 (72.3)
	Counselling skills	8	7.6 (7.3–8.0.3.0)	16 (24.6)	53 (81.5)
	Professionalism	7	7.7 (7.3–8.0.3.0)	15 (23.1)	54 (83.1)
	Organisation and efficiency	7	7.4 (7.0–7.8.0.8)	14 (21.5)	49 (75.4)
	*Overall clinical competence*	*7*	*7.6 (7.3–7.9)*	*16 (24.6)*	*53 (81.5)*

*Mini-CEX; Mini Clinical Evaluation Exercise*,* 95% CI; 95% Confidence interval*

*Assessment categories: 1–3 = unsatisfactory,* 4–6 = satisfactory*,* 7–9 = superior*

## Discussion

In the current study we have evaluated components of construct validity of the Mini-CEX instrument as an assessment tool in the context of clinical placement in dietetics education. Clinical supervisors were able to discriminate between levels of clinical competence of portrayed master students in dietetics in scripted consultations using Mini-CEX scores. They demonstrated highest agreement with the reference scores established by an assessment panel on the unsatisfactory and superior student performance.

Percent agreement has been criticised for not adjusting for chance agreement and therefore overestimating true agreement, but still remains a popular measure of interobserver agreement [41]. Higher percent agreement was demonstrated in the current study than in a similar study by Cook et al. [45]. In their study, participants received training in a workshop before rating 16 scripted and unscripted videotaped encounters in a pretest-post-test design, and they still only reached a percent agreement of 53–55%. The known overestimation of true agreement will usually be apparent by higher agreement in the middle of the rating scale than at the extreme ends [46]. In our results, agreement was highest in the extreme ends, in favour of true agreement on unsatisfactory and superior performance. The Krippendorff’s alpha, on the other hand, considers chance-corrected agreement and often results in an underestimation of true interrater reliability [41]. Our alpha coefficient indicated substantial agreement despite its known tendency for conservative estimates. Together with our use of multiple statistical approaches to evaluate the validity of the instrument, this strengthens the validity argument [47].

In our study, we attempted to isolate and demonstrate three distinct components of clinical competence when developing the video scripts and the videos (*clinical communication skills*,* clinical reasoning*, and *professionalism*). Our results confirm the difficult task it is to succeed in doing this [34]. The consistent high correlations we found between the competency scores within each video likely underpins the presence of a halo effect, as demonstrated in other studies [48–50]. The halo effect refers to an assessor’s tendency to provide ratings on a global impression of competence of the student instead of differentiating between dimensions of competence [51]. This tendency can lead to consistently high or consistently low scores by an assessor and failure to identify specific aspects for improvement for someone receiving high scores, or aspects worthy of praise for someone receiving low scores. Diverging explanations for such high correlations are found in the literature. Cook et al. [52] raise the possibility that an assessor only assesses one overall clinical competence when utilising Mini-CEX, and that one must therefore be careful when interpreting the scores to represent distinct aspects of clinical competence. Such a concern is particularly warranted if the instrument is used for summative purposes, which would be contrary to intended use. Conversely, Hawkins et al. [53] stated in their review that another possible explanation for high correlations between competency scores is that they reflect true relationships between the underlying constructs, as several of them are indeed expected to be highly correlated.

Previous studies [17, 24, 32] have shown that repeated encounters with the Mini-CEX are desired to accurately assess student performance, pointing to how an increase up to ten encounters gains consistency by reducing the 95% confidence intervals (CI) of the Mini-CEX scores [17] or increasing generalisability coefficients [32]. In an attempt to include repeated encounters when assessing validity, Holmboe et al. [24] and Arora et al. [34] developed multiple videos for each case with the same student depicting different levels of performance for selected competencies. Presumably, assessors could then calibrate their scores for each student, as each student would be observed several times. Though multiple videotapes provide for a wider selection of data points in the validations, this is built on an artificial premise as supervisors do not observe the same student perform the same consultation on several occasions in the clinic. In favour of our study design with fewer videotapes and many assessors, Margolis et al. [48] conclude that many raters assessing the same performance, though on few occasions, *enhances* score stability; the variance and measurement error when one assessor scores the same student’s performance on many occasions is larger than if many assessors score one student’s performance only once, as in our study.

Our data, with highest percent agreement on the unsatisfactory and superior performance, does not support the theory of assessors tending to avoid scale extremes, though other studies have observed this [49]. The participants agreed the most on the unsatisfactory performance of the student in the Overweight video, contrary to Palermo et al. [3] which described how (novice) supervisors generally find it easier to assess students performing at high levels than low. Participants had been informed that in an authentic clinical setting, assessing a student performance as unsatisfactory at this time of placement would be the equivalent to voicing their concern to the university. The fact that they agreed the most on the level of student performance in the Overweight video, shows that they did not demonstrate the typical behaviours of reluctance to failing the underperforming student – known as “failure to fail” – despite being informed of the high stakes [54]. The observed consensus on what constitutes unsatisfactory performance is an important support for both validity and utility of the instrument. Being able to identify very poor, maybe even harmful, clinical performance is of utmost importance, and according to our data, Mini-CEX is a promising instrument to mitigate the risks of overly lenient judgements, or failure to identify students requiring additional support. It is worth noting that the low scores could be a consequence of the video format, which has been shown to lower the threshold for assigning low scores compared to direct observation and assessment in authentic situations [53]. Already in the earliest years of developing and utilising Mini-CEX, Norcini et al. [16] emphasised the importance of increased number of encounters for borderline competent students, due to the educational value of the feedback conversation following assessment, where students get an opportunity for learning and self-correction [16].

The clinical competence of the student in the Malnourishment video, which was scored as overall satisfactory by the assessment panel, received scores ranging from unsatisfactory to superior by our participants. The large divergence in Mini-CEX scores on what was intended to depict an acceptable level of clinical competence, is an interesting observation, but it is not new. Many mechanisms can give rise to rater variability in assessment of the same performance, including differences in focus, differences in emphasis, comparisons to past observations and uncertainty of the underlying criteria [55]. Assessors have been shown to find it more challenging to evaluate students who perform at an average level, compared to those who are either clearly struggling (unsatisfactory performance) or clearly excelling (superior performance) [56]. Several studies in dietetics, though with different assessment instruments, have demonstrated how assessor ratings are inherently subjective, with large variation and primarily reflect assessors’ interpretations of student performance, not objective measures of actual performance [57, 58]. Critical dialogue between the assessors on the criteria of clinical competence and (video) performance of dietetics students has been found to promote more holistic judgements and can prompt many assessors to revise their assessment ratings afterwards [57, 58]. Such critical dialogue is similar to what the assessment panel in our current study had, when reaching consensus scores used for reference. This further underscores the importance of multiple Mini-CEX encounters with different assessors, and also of building a strong community of clinical supervisors dedicated to enhancing supervision, assessment, and feedback practices amongst them [20]. It also advocates for adopting a more holistic approach to assessment design, with continual assessment for learning, and meticulous gathering of information from multiple sources before making any high-stake decisions [14].

It has been proposed by several scholars to add descriptive anchors to the Mini-CEX for what constitutes both unsatisfactory and satisfactory performance for each competence, in addition to the descriptions for superior performance which is included in most Mini-CEX versions already [34, 59]. Such alterations could enable supervisors to provide more well-substantiated scores for each competency and maintain a clear distinction between them. This could in turn counteract the large divergence in scores between assessors for satisfactory performance, and possibly the halo effect as assessors familiarise with the instrument and competencies.

Our results demonstrated a sample population which utilised the Mini-CEX seemingly well despite having little previous experience with the instrument and little formal training in supervision. This could indicate that comprehensive assessor training in the direct observation and assessment using Mini-CEX might not be necessary for a valid score assessment to take place. In the assessment of clinical competence, both the impact and optimal form of assessor training are debated, prompting calls for further investigations to best support successful Mini-CEX implementation [60]. In a review, Kogan et al. [61] promote the importance of training to correctly assess student performance in direct observation but conclude that if training is brief, it is ineffective. Quantitative and qualitative studies have shown that Mini-CEX assessment training can improve rater consistency, boost assessor confidence and competence understanding, and strengthen the narrative feedback to complement numerical ratings [3, 45, 62, 63]. Somewhat conversely, Lennie et al. [5] reported how it might be sufficient for a department of supervising clinical dietitians to simply utilise a specific assessment tool (e.g. Mini-CEX) over time to quantitatively assess students’ competence levels with increasing consistency amongst them. Others warn that relying on a single instrument may create a false sense of sufficiency [20], emphasising that assessors – through their expert judgment – are the true tools in observational assessment and need extensive training [13]. While it may not directly improve Mini-CEX score accuracy, pedagogical training and increased confidence can still encourage clinical dietitians to take on supervisory roles and, above all, enhance the feedback component.

The intended main purpose of using Mini-CEX in formative assessment is to provide a foundation for an educational feedback conversation between supervisor and student. When such conversations are done well and repeated over time, students are empowered to make the most of the feedback information they receive, process and act on it in subsequent encounters [20]. The use of an assessment tool with existing validity evidence has been recommended strongly as the basis for feedback following direct observation of students. It is emphasised that quantitative ratings must then be complemented with narrative feedback for a meaningful interpretation of scores to facilitate learning [20]. Studies on the feedback session following direct observation and Mini-CEX assessment have been performed in both undergraduate and residency programs within medical education [64, 65], but has to our knowledge not been performed in the dietetics education context. Such a study would help us gain insight into what further training is needed for both faculty and students to optimise the educational impact of the process, as teaching of feedback skills has been recommended as part of both student and faculty development programs [2].

There has been a shift towards more formative assessment in HPE, especially in the context of a programmatic assessment design [14]. In such a design, emphasis is placed simultaneously on assessment for learning (low-stake) and on optimising assessment for judgement (high-stake) [13]. Consequently, concerns have been raised towards the blurry lines which then arise between assessment and feedback, where the same supervisor is expected to act as both a coach facilitating learning and a judge making decisions [66]. This is thought to hinder quality feedback processes, as students often perceive any observation and assessment as high-stake and can become reluctant to educational feedback [66]. To counteract this, careful consideration of assessment and feedback design is vital, where a direct observation and assessment using Mini-CEX can be one of many points of assessment and sources of feedback. The intent of every observation, assessment and instrument which is used must be clearly articulated to the student, and clinical educators must be supported [66].

Based on our findings and current literature [12, 15], we propose two key focus areas for further development of assessment and feedback practices, with the aim of optimising student learning outcomes in clinical placements:

Firstly, we recommend assessor training which mainly addresses pedagogical and qualitative aspects regarding the feedback conversation following assessment with an instrument like Mini-CEX. Such training should encompass understanding of and training in new paradigm feedback practices and evidence-based strategies for effectively facilitating them [20, 67]. Secondly, we believe students and assessors will benefit greatly from the latter engaging in critical dialogue and group discussions on what constitutes clinical competence in dietetics. This will enable clinical supervisors to make more consistent judgements of borderline and mid-level performing students in accordance with established consensus. These two focus areas align well with a programmatic assessment approach, where multiple sources of quantitative and qualitative evidence contribute to a comprehensive evaluation of student competence [15]. They will also facilitate longitudinal development and holistic judgment against established competency standards, without relying on just one individual assessment or assessment instrument. Such dialogue and training in facilitation of feedback processes can be vital to cultivate a community of practice for the supervision, assessment and feedback for students amongst clinical dietitians when balancing their roles as clinicians and educators [5].

### Strengths and limitations

#### *Strengths*

In this study, we have assessed and supported the construct validity of Mini-CEX scores by assembling evidence from multiple sources, in accordance with contemporary understandings of validity [47], and more comprehensively than in several prior validation studies [24, 25]. Previous studies in other contexts that have adopted similar videotape-based methods, have included 40–52 individual raters of videos [24, 34, 49]. Hence, our successful recruitment of a sample population of 65 participants strengthens the results. The study design with its use of a rigorous video format is a robust way of making sure variability in the collected assessment scores is not due to variability in student performance. Another strength with the current study is the use of an assessment panel to discuss and agree upon the performance level of the portrayed students in the videos. We have seen several validation studies of Mini-CEX in different contexts using scripted performances, often videos, but not including an assessment panel. A similar approach involving assessor discussions before finalising their ratings on dietetics student (video) performance has been undertaken, though with a different assessment instrument and not to evaluate validity [58]. Scripted videos may be influenced by acting skills, actor dynamics, and recording instructions, which likely affect how clearly intended performance levels are conveyed. By incorporating the step of an assessment panel, we have accounted for several of these factors and emphasised that the videos – rather than the prepared scripts – offer the most authentic representation of the portrayed students’ competence. The assessment panel ensures that reference scores are grounded in actual, rather than scripted, performance.

#### *Limitations*

The investigation of the use of Mini-CEX was limited to a one-time assessment of each student’s performance in patient consultation, and only in the video format, contrary to the intended use of the instrument. Though we acknowledge that the video format introduces an artificial element to the interaction which assumably influences assessors’ judgment, having this many assessors score the same performances would be unfeasible without use of video recordings. The current study was limited to exclusively quantitative aspects of the Mini-CEX. Further exploration of qualitative dimensions – such as participants’ perceptions of the instrument’s feasibility and further implementation potential – would have provided valuable insights for stakeholders.

### Future perspectives

The present study on construct validity of Mini-CEX assessment in the dietetics context is an important foundation for further research on the qualitative aspects of students’ feedback processes in clinical placement. As stated in a review by Pelgrim et al. in 2011 [68] *“(…) educational effects are a neglected area of assessment research*,* which should be given much greater priority in future research”*, this remains true to date. Learning and improvement of clinical skills, being the main objective of formative assessment, is an area which still warrants further research. A central aspect for future investigation is how direct observations and assessment using Mini-CEX are followed by feedback conversations and how the scores are then used for feedback purposes. To gain deeper insight into the full process of formative assessment in clinical placement, an investigation of Mini-CEX in the context of multiple encounters, monitoring progress over time in an authentic environment is one feasible example worth pursuing.

To ensure that valid inferences on clinical competence can be drawn from Mini-CEX scores, further evaluation of the validity argument in the context of dietetics education is warranted. Such evaluation should include assembling evidence on correlations between Mini-CEX scores and other instruments’ scores as well as intended or unintended consequences of Mini-CEX assessment for the student [33].

Future studies should also investigate the use of Mini-CEX between peers in peer placement settings, as these are common in HPE [59, 69, 70], as well as electronic versions of the paper-based Mini-CEX, so-called personal digital assistants (PDAs), and what these might add to the process of formative assessment. Such electronic versions were not yet common in our study setting but have shown great promise in empowering students to take responsibility for their own learning process, while simultaneously holding supervisors accountable regarding the feedback component of the assessment [71].

## Conclusions

The present study demonstrates that clinical dietitians sufficiently discriminate between portrayed master students’ level of clinical competence when using the Mini-CEX instrument, in agreement with an experienced assessment panel. Our findings support the construct validity of the instrument as a tool in the formative assessment of dietetics students in clinical placement. Furthermore, our study indicates that Mini-CEX is an instrument that can be readily translated and adapted across national, disciplinary, and contextual boundaries. Consensus amongst assessors and training in how to provide feedback is needed to ensure successful implementation. When a valid Mini-CEX assessment based on direct observation of students’ performance is used as a foundation for specific feedback, it can aid the learning and professional development for students during outplacement.

## Supplementary Information

Supplementary Material 1.

Supplementary Material 2.

## Data Availability

The datasets used and analysed during the current study are available from the corresponding author on reasonable request.

## Abbreviations

CEX: Clinical evaluation exercise
CI: Confidence interval
ECTS: European Credit Transfer and Accumulation System
EFAD: European Federation of the Associations of Dietitians
HEI: Higher Education Institution
HPE: Health Professions Education
ID: Identification
PDA: Personal digital assistant
