# Supplementary material for: Validating an assessment and feedback instrument for use in dietetics education: construct validity of the mini clinical evaluation exercise (Mini-CEX)
Source: BMC Med Educ. 2025 Dec 12;25:1693. doi: 10.1186/s12909-025-08255-8 (PMC12699876; doi:10.1186/s12909-025-08255-8)
Supplement: Supplementary file 1 — Supplementary Material 1. [file 12909_2025_8255_MOESM1_ESM.docx]

Background survey Mini-CEX

Following, are six short multiple-choice questions to give us background information about you and your experience with supervision and Mini-CEX.

| **Question** | **Answer** |
| --- | --- |
| Enter the ID number you have been assigned for the study (number between 1-70): | Open field |
| How old are you? | < 25 years  26-35 years  36-45 years  46-55 years  > 55 years |
| What is your gender? | Female  Male  Other |
| How long have you been working as a clinical dietitian? | < 2 years  2-4 years  5-7 years  8-10 years  > 10 years |
| How many master students in nutrition have you supervised in clinical placement? | None  1-3 students  4-6 students   - 1. students   > 10 students |
| Have you taken courses or education in clinical supervision? | Yes No |
| Do you have previous experience with the Mini-CEX form? | No experience  Some experience  Extensive experience |

Thank you for your participation!
