# Supplementary material for: Validating an assessment and feedback instrument for use in dietetics education: construct validity of the mini clinical evaluation exercise (Mini-CEX)
Source: BMC Med Educ. 2025 Dec 12;25:1693. doi: 10.1186/s12909-025-08255-8 (PMC12699876; doi:10.1186/s12909-025-08255-8)
Supplement: Supplementary file 2 — Supplementary Material 2. [file 12909_2025_8255_MOESM2_ESM.pdf]

Mini-CEX til vurdering av ernæringsstudent

Generell informasjon

Dato: ………/………./……….

Setting [ ] Poliklinikk [ ] Sengepost [ ] Primærhelsetjenesten
[ ] Voksen [ ] Barn

Diagnose: ………………………………….

Ranger studenten etter det du mener kan forventes av han/henne

	Utilfredsstillende			Tilfredsstillende			Fremragende			Ikke obs
Klinisk samtale	1	2	3	4	5	6	7	8	9	i.o.
Innhenting av opplysninger	1	2	3	4	5	6	7	8	9	i.o.
Kliniske vurderinger og beslutninger	1	2	3	4	5	6	7	8	9	i.o.
Rådgivningsferdigheter	1	2	3	4	5	6	7	8	9	i.o.
Profesjonalitet	1	2	3	4	5	6	7	8	9	i.o.
Organisering og effektivitet	1	2	3	4	5	6	7	8	9	i.o.
Alt i alt	1	2	3	4	5	6	7	8	9	

Styrker

Forslag til forbedringer

Signatur veileder

Signatur student

Forklaringer til skjema

Rangering

Utilfredsstillende – studenten presterer dårligere enn forventet på dette nivået i utdanningen.

Tilfredsstillende – studenten presterer som forventet på dette nivået i utdanningen.

Fremragende – studenten presterer over forventet på dette nivået i utdanningen.

Utdyping av kategoriene i Mini-CEX

Klinisk samtale
Bruker pasientsentrert tilnærming og utforsker pasientens ernæringstilstand, sykehistorie, bekymringer, forventninger, oppfatninger og livssituasjon. Responderer på verbale og non-verbale tegn. Veksler mellom å lytte og snakke. Bruker åpne og lukkede spørsmål strategisk. Etterprøver problemforståelsen.

Innhenting av opplysninger
Utfører en strukturert kartlegging og/eller undersøkelse i samarbeid med pasienten. Samtaler med pasienten om hva som undersøkes underveis. Viser hensyn til pasientens komfort og bekvemhet.

Kliniske vurderinger og beslutninger
Relaterer ernæringsstatus, anamnese og andre opplysninger til hverandre. Utøver rasjonell bruk av undersøkelser. Bruker forskningsbasert kunnskap, egne erfaringer og pasientens kunnskap, verdier og behov som beslutningsgrunnlag. Bruker godt skjønn i vanskelige avveielser.

Rådgivingsferdigheter
Etablerer en terapeutisk allianse med pasienten. Vurderer aktuell ernæringsbehandling sammen med pasienten. Styrker pasientens evne til å ta et informert samvalg. Fremmer etterlevelse og livsstilsendring på pasientens premisser. Unngår utilbørlig press på pasient eller pårørende i beslutningsprosesser.

Profesjonalitet
Opptrer høflig og tilpasser egen væremåte til situasjonen. Viser respekt og omsorg for pasient og pårørende. Oppdager og håndterer følelsesmessige reaksjoner. Samarbeider med kollegaer. Ivaretar taushetsplikten.

Organisering og effektivitet
Arbeider effektivt. Balanserer tidsbruken i innsamling av klinisk informasjon. Bidrar til å strukturere og gjennomføre kollegaers og pasientens aktivitet. Forvalter fellesskapets ressurser på en fornuftig måte.
